# Failure to apply standard limit-of-detection or limit-of-quantitation criteria to specialized pro-resolving mediator analysis incorrectly characterizes their presence in biological samples

**DOI:** 10.1038/s41467-023-41766-w

**Published:** 2023-11-09

**Authors:** Valerie B. O’Donnell, Nils H. Schebb, Ginger L. Milne, Michael P. Murphy, Christopher P. Thomas, Dieter Steinhilber, Stacy L. Gelhaus, Hartmut Kühn, Michael H. Gelb, Per-Johan Jakobsson, Ian A. Blair, Robert C. Murphy, Bruce A. Freeman, Alan R. Brash, Garret A. FitzGerald

**Affiliations:** 1 Systems Immunity Research Institute, School of Medicine, Cardiff University, CF14 4XN Cardiff, Wales UK; 2 Chair of Food Chemistry, Faculty of Mathematics and Natural Sciences University of Wuppertal, Gausstraße 20, 42119 Wuppertal, Germany; 3 Division of Clinical Pharmacology, Vanderbilt University, 502A Robinson Research Building, Nashville, TN 37232-6602 USA; 4 Medical Research Council Mitochondrial Biology Unit, University of Cambridge, Cambridge, CB2 0XY UK; 5 School of Pharmacy and Pharmaceutical Sciences, Cardiff University, Cardiff, CF10 3AT UK; 6 Institute of Pharmaceutical Chemistry, Goethe University Frankfurt, Max-von-Laue-Str. 9, 60438 Frankfurt, Germany; 7 Department of Pharmacology and Chemical Biology, University of Pittsburgh, Pittsburgh, PA 15261 USA; 8 Institute of Biochemistry, University Medicine Berlin – Charité, Berlin, Germany; 9 Department of Chemistry, University of Washington, Seattle, WA 98195 USA; 10 Rheumatology Unit, Dep. of Medicine, Solna, Karolinska Institutet & Karolinska University Hospital, Stockholm, Sweden; 11 Department of Systems Pharmacology and Translational Therapeutics, Perelman School of Medicine, University of Pennsylvania, Philadelphia, PA 19104 USA; 12 Department of Pharmacology, University of Colorado Denver, Aurora, CO 80045 USA; 13 Department of Pharmacology, Vanderbilt University, Nashville, TN 37232 USA; 14 Institute for Translational Medicine and Therapeutics, Smilow Center for Translational Research, University of Pennsylvania, Philadelphia, PA 19104 USA

**Keywords:** Lipidomics, Lipids

**arising from** E.A. Gomez et al. *Nature Communications* 10.1038/s41467-020-19176-z (2020)

Specialized pro-resolving mediators (SPM) derived from oxygenation of long chain polyunsaturated fatty acids (PUFA) were originally described by Serhan and colleagues and have been proposed as mediators of inflammation resolution. Families of SPM described in the literature include lipoxins, resolvins, maresins, protectins and their peptide conjugates. Gomez and co-authors reported that levels of plasma SPM from patients with early rheumatoid arthritis predict response to biologic therapy after 6 months. SPM were measured in this study using liquid chromatography tandem mass spectrometry (LC-MS/MS). On reviewing the methods, supplementary analytical data, and the online peer review file, we note serious concerns, regarding both analytical methods and experimental conclusions. Application of this flawed methodology to SPM analysis brings into question the very occurrence of many of these lipids in biological samples, their proposed impact on inflammatory processes, and claims of their utility as biomarkers.

In Gomez et al.^1^, the authors do not use signal-to-noise ratio (S/N) for determining limit of detection (LOD), instead they cite the following criteria for identification and quantification of lipids: (1) presence of a peak with a minimum area of 2000 counts. (2) matching retention time to synthetic or authentic standards with maximum drift between the expected retention time and the observed retention time of 0.05 s. (3) ≥4 data points, and (4) matching of at least 6 diagnostic ions to that of reference standard, with a minimum of one backbone fragment being identified in representative samples.

This means that established analytical guidelines from multiple agencies, of calculating S/N ratio and using this to set LOD and limit of quantitation (LOQ) are not applied. Full information on S/N, and its application is in Supplementary Text. Noting this, we assessed their method experimentally, focusing particularly on criteria 1 and 4, above.

(1) Presence of a peak with a minimum area of 2000 counts—A mass spectrometer calculates chromatographic peak area under the curve as counts per second (cps). However, we found that >2000 cps can be recorded using HPLC-grade methanol blanks for at least 5 oxylipins (Fig. 1, Supplementary Figs. 1 and 2A). Furthermore, background noise in tissue samples will be higher than standards and varies significantly between detection channels. This renders this non-standard approach misleading.

**Fig. 1:**
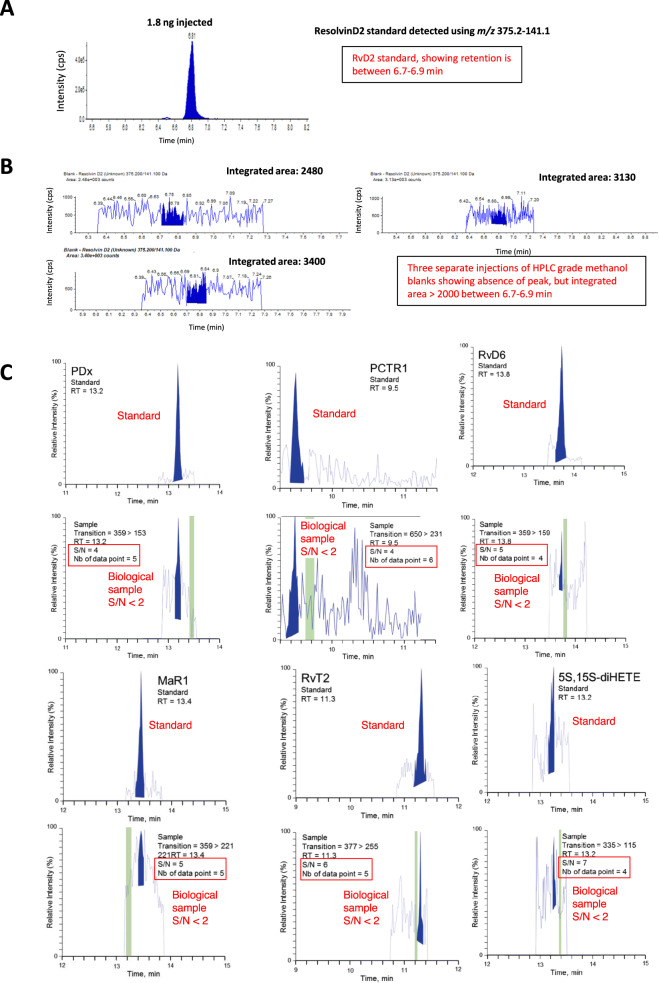
Analysis of methanol blanks shows integrated peak areas >2000 cps for RvD2, while flawed S/N analysis shows false positives for several SPM in Gomez et al. **A** Example chromatogram from 1.8 ng RvD2 standard analyzed using LC-MS/MS as described in Methods. **B** Three separate analyses of a methanol injection, in the region where RvD2 elutes showing the areas where the signal was integrated. **C** Chromatograms taken directly from the Supplementary information of Gomez et al^1^. The chromatograms are representative of many from Supplementary information, in Gomez et al^1^, showing the authentic standard on top with analysis of the biological sample immediately below. In BLUE are the peaks areas computed by Gomez et al^1^ and the green strips are the regions the authors used to calculate S/N ratios of 4, 4, 5, 7, 6 and 5, reading clockwise from the top left panel. In Red, we have added to the originals for clarification: labels of Standard, Biological Sample, a box around the original S/N, and estimations of S/N < 2, considering the SPM signal and the entire available baseline.

We reviewed chromatograms in Gomez et al.^1^ and show six with unsatisfactory S/N, representing false positives: PDx, PCTR1, RvD6, Mar1, RvT2, 5 S,15S-diHETE (Fig. 1C). In these and others (e.g., RvD5, 17R-RvD1, 17R-RvD3, PCTR2, 22-OH-MaR1, 14-oxo-MaR1, 7S,14S-diHDHA, MCTR2, RvT4, RvD5(n-3 DPA), PD1(n-3 DPA), MaR1(n-3 DPA), LXA4, 15R-LXB4, and TXB2), the S/N ratio is <5, providing no basis for quantitation. The reported SPM, assessed for S/N using the standard approach is shown (Supplementary Table 1). Only 16 of 55 were acceptable (S/N > 5), with another 5 borderline (S/N > 3). In many there is no discernible peak at the expected retention time (blue region), indicating clear integration of non-analyte components of noise (Fig. 1C). During revision of this article, raw data was not provided to the journal, due to intellectual property and patient confidentiality claims, thus limiting independent analysis of MS data. In their rebuttal^2^, Dalli and Serhan apply a method called Relative-Noise-Concept using an algorithm from Sciex. Large S/N values were generated from datasets containing no visible peak (Supplemental Fig. 2 in ref. ^2^). Software calculating S/N should never be utilized in this manner, as this generates false positive data. Furthermore S/N should not be determined after smoothing^3^.

(4) Matching of at least 6 diagnostic ions to that of reference standard, with a minimum of one backbone fragment being identified in representative samples—In standard practice, for a positive match the MS/MS spectra of standard and sample should be visually similar when recorded on the same instrument using the same parameters. The dominant product ions should be present in both with a similar pattern of relative abundance. In Gomez et al., the spectra of RvD1 standard and sample are weak and noisy; there are different relative abundances of ascribed “diagnostic” ions. The large prominent RvD1 ion that should be at *m/z* 215 is not distinguishable from background (Fig. 2A). A clean standard is shown for comparison, obtained on a 6500 QTrap (Fig. 2B). Overall, many product ion spectra in the Supplement do not match standards and are of extremely poor quality. Thus, their use for identification purposes is incorrect and misleading and these data don’t support the presence of SPM in the samples. When lipids are detected close to, or are below LOD in a biological sample, acquired spectra would not visually compare well with standards. If poor quality MS/MS spectra are used, the approach is fundamentally flawed, even if software erroneously claims a close match (Supplementary Fig. 4 in ref. ^1^).

**Fig. 2:**
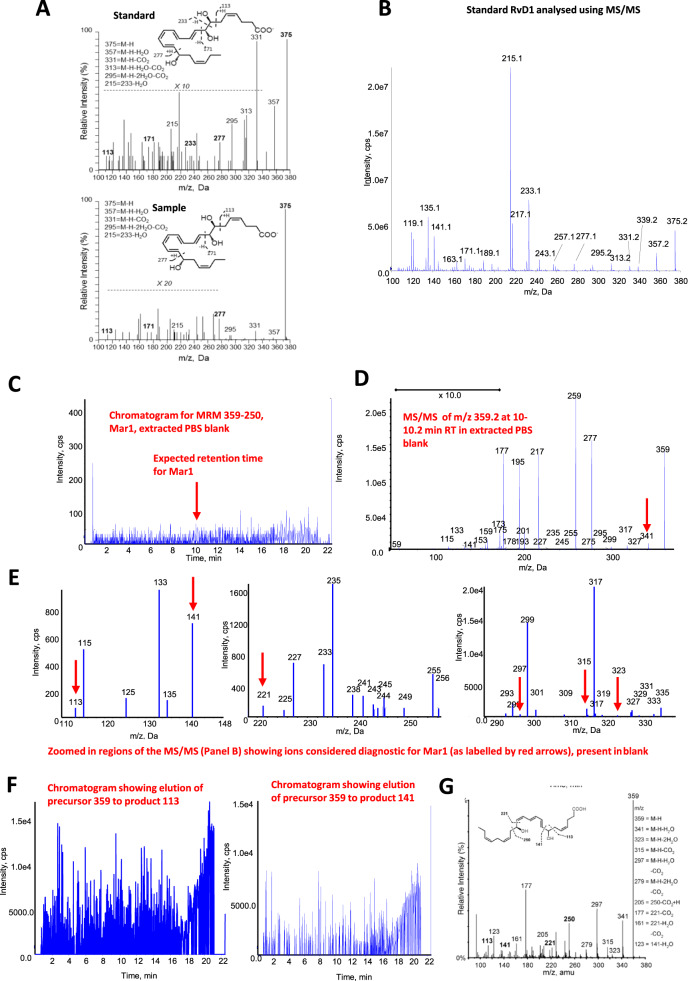
MS/MS of RvD1 do not match between standard and sample, and an extracted buffer blank shows absence of peak, but several detectable “diagnostic” ions for Mar1, with some being detected throughout the entire run. **A** Screenshot of Supplementary Fig. 1A showing MS/MS of standard and sample, from Gomez et al. **B** MS/MS of an RvD1 standard generated in one of our laboratories. **C** Chromatogram, monitoring for Mar1 at *m/z* 359–250. **D** MS/MS at 10–10.2 min, where the Mar1 standard elutes, showing isolation and fragmentation of ion at *m/z* 359. **E** Zoomed in regions of centroid spectrum showing background ions contain several “diagnostic” ions for Mar1, as labelled by red arrows. **F** Ions at *m*/*z* 113 or 141 that are detected following fragmentation of m/z 359 are detected throughout the chromatographic run. Panel G. Figure showing first report of Mar1 MS/MS spectrum from^12^. **G** Reproduced from Serhan et al.^12^, the first report of maresin-1 (Mar1) as a novel metabolite of DHA.

We next looked at MS/MS data in papers cited by Gomez in support of the method^4–11^. Three report RvD1 and RvD3 in serum or synovial samples^4,6,11^. There is a lack of similarity between spectra from these different studies. Low abundance ions, many indistinguishable from background are often labelled. In one study, there are 2 MS/MS spectra for RvD3, in mouse paws or human serum (Figures 1, 3, respectively^4^) that differ considerably from each other and from spectra in the other articles. Importantly, these bear no resemblance to the MoNA reference spectrum (https://mona.fiehnlab.ucdavis.edu/spectra/display/IA000269), which has ions at *m/z* 69, 95, 115, 137 and a large prominent ion at 147.

Today’s MS platforms including the Sciex QTraps, are highly sensitive, and capable of detecting low level noise signals from the laboratory, solvents, or electronics. To demonstrate this, phosphate buffered saline was processed using solid phase extraction and analyzed using LC–MS/MS while acquiring enhanced product ion (EPI) spectra for Mar1. No chromatographic peak was seen (Fig. 2C). However, at the retention time for Mar1 (and throughout the entire run), the instrument isolated a background noise signal at *m/z* 359 which could be fragmented (Fig. 2D). Zooming in, following conversion of the spectrum from profile to centroid, many background noise ions are seen (*m/z* 113, 141, 221, 297, 315, 323, 341) that match the “diagnostic” ions shown in Supplementary Fig. 1P of Gomez^1^ (Fig. 2D, E). Furthermore, low intensity noise at *m/z* 113 or 141 derived from *m/z* 359 are visible across the full chromatogram (Fig. 2F). A literature reference spectrum for Mar1 is reproduced for reference (Fig. 2G)^12^. The product ion of *m/z* 250, deemed “diagnostic” for Mar1^12^ is absent in both the sample and standard MS/MS spectra presented by Gomez et al, in Supplementary Fig. 1P^1^. Similarly, analysis of extracted PBS for several putative resolvin mass transitions showed ions at the expected precursor masses, which could in turn generate variable signals of “diagnostic” product ions from noise (Supplementary Figs. 2B, C and  3). Complex lipid extracts from tissue would have far higher levels of background noise to contribute to MS/MS. This data affirms that high sensitivity mass spectrometers can generate extremely poor quality noise MS/MS spectra from blanks, which have been analyzed incorrectly to infer the presence of SPM^1,4–11^.

In summary, methanol or buffer blanks can generate integrated areas in excess of 2000 cps, as well as poor quality MS/MS data that can erroneously suggest SPM precursor and product ions that are reported in datasets from Gomez et al^1^. The method used by Gomez^1^ and cited articles^4–11^ is flawed, artifactually detecting lipids where none exist. Since this is the analytical approach most commonly used for SPM analysis, the evidence for the presence of SPM in biological matrices and their inferred role in inflammation resolution needs re-evaluation.

## Methods

See Supplementary Methods.

### Reporting summary

Further information on research design is available in the Nature Portfolio Reporting Summary linked to this article.

### Supplementary information

Supplementary Information

Reporting Summary

## Data Availability

The data which support the figures and other findings within this paper are available from the corresponding authors upon request.
