# Supplementary material for: Failure to apply standard limit-of-detection or limit-of-quantitation criteria to specialized pro-resolving mediator analysis incorrectly characterizes their presence in biological samples
Source: Nat Commun. 2023 Nov 9;14:7172. doi: 10.1038/s41467-023-41766-w (PMC10636151; doi:10.1038/s41467-023-41766-w)

Supplementary Information

Supplementary main text

Signal/noise (S/N) criteria is the approved methodology set by the International Conference on Harmonisation (ICH), the European Pharmacopoeia (Ph. Eur), the International Organisation for Standardisation (ISO), the European Medicines Agency (EMA), the Food and Drug Administration (FDA), the United States Pharmacopeia (USP), the International Union of Pure and Applied Chemistry (IUPAC), and the World Health Organisation (WHO)[1-10]. While validated assays have recommended S/N of around 10 for LOQ, and 3:1 for LOD[1], the practice of many academic research labs has been to use around 5:1 for LOQ. Consistent with this, the FDA requires that the analyte response at the LOQ should be ≥ 5 times the analyte response of the zero calibrator[11].

The Serhan/Dalli method is described in more detail in two online protocols, which confirm the lack of S/N criteria being applied to set LOD/LOQ[12,13]. Worryingly, the integration of peaks with S/N <3 is actively encouraged in one protocol[13]. In a second protocol, the use of 2000 cps as baseline is recommended, and both describe how to confirm the presence of lipids using 6 MS/MS "diagnostic ions" [12,13].

Supplementary Methods

Newly opened SPE cartridges (Waters, Sep-Pak C18, 6 mL capacity, 500 mg) were conditioned with 5 mL of methanol (Fischer, HPLC grade) followed by 10 mL of Ultrapure water (Cayman). 5 mL of phosphate buffered saline was then loaded and columns washed with 10 mL of Ultrapure water. 3 mL ethyl acetate (Sigma-Aldrich, LC-MS grade) was used to elute oxylipins. This was evaporated under vacuum and samples re-dissolved in methanol, before being analysed using LC-MS/MS as described. LC-MS/MS was performed on a Nexera liquid chromatography system (Nexera X2, Shimadzu) coupled to a 6500 QTrap mass spectrometer (AB Sciex). Liquid chromatography was performed at 45 °C using a Zorbax Eclipse Plus C18 (Agilent Technologies) reversed phase column (150 × 2.1 mm, 1.8 µm) at a flow rate of 0.5 mL/min over 22.5 min. Mobile phase A was (95 % HPLC water/5 % mobile phase B; v/v and 0.1 % acetic acid) and mobile phase B was acetonitrile/methanol (800 ml + 150 ml; and 0.1 % acetic acid). The following linear gradient for mobile phase B was applied: 30 % for 1 min, 30 - 35 % from 1 to 4 min, 35 – 67.5 % from 4 to 12.5 min, 67.5 – 100 % from 12.5 to 17.5 min and held at 100 % for 3.5 min, followed by 1.5 min at initial condition for column re-equilibration. Injection volume was 5 µL. Lipids were analyzed in monitoring (MRM) mode with scheduling (55s) for the baseline integration experiment. Ionization was performed using electrospray ionization in the negative ion mode with the following MS parameters: temperature 475 °C, N_2 gas, GS1 60 psi, GS2 60 psi, curtain gas 35 psi, ESI voltage -4.5 kV. Cycle time was 0.4 s. For MS/MS analysis, enhanced product ion mode was used with dynamic fill time. Data were integrated using Analyst software. Data showing integrated windows are shown as screenshots, while MS/MS analysis was copied into PowerPoint for minimal processing (linewidths, font sizes only) with no alterations to chromatographic or MS/MS data. MS/MS is presented as profile or centroid as described in Figure Legends. Oxylipin

standards were from Cayman Chemical. S/N ratio was manually calculated by measuring peak height (down to the midpoint of the noise) and noise height (full height across a clearly representative area of baseline), and dividing signal by noise. An example is shown here[14].

Supplementary Figure and Table Legends

Supplementary Figure 1. Further examples where baseline noise integration generates signals higher than 2000 cps. Panel A. Example chromatogram from LTB3 standard analysed using LC-MS/MS as described in Methods. Three separate analyses of a methanol injection, in the region where LTB3 elutes showing the areas where the signal was integrated. Panel B. Example chromatogram from 8-HETE standard analysed using LC-MS/MS as described in Methods. Three separate analyses of a methanol injection, in the region where 8-HETE elutes showing the areas where the signal was integrated. Panel C. Example chromatogram from 15-HETE standard analysed using LC-MS/MS as described in Methods. Two separate analyses of a methanol injection, in the region where 15-HETE elutes showing the areas where the signal was integrated.

Supplementary Figure 2. Examples of baseline noise integration and the presence of false "diagnostic" ions in MS/MS from extracted buffer blanks. Panel A. Example chromatogram from 5-HETE standard analysed using LC-MS/MS as described in Methods. Analysis of a methanol injection, in the region where 5-HETE elutes showing the areas where the signal was integrated. Panel B. Chromatogram, monitoring for RvD1 at *m/z* 375-215 in standard and blank. Panel C. MS/MS at 7.2-7.4 min, where the RvD1 standard elutes, showing isolation and fragmentation of ion at *m/z* 375. Zoomed in regions of centroid spectrum showing background ions incorrectly identified as "diagnostic" ions for RvD1, as labelled by red arrows.

Supplementary Figure 3. The presence of putative "diagnostic" ions in MS/MS from extracted buffer blanks Panel A. Chromatogram, monitoring for RvD5 at *m/z* 359-199 in standard and blank. Panel C. MS/MS at 9.9-10.1 min, where the RvD5 standard elutes, showing isolation and fragmentation of ion at *m/z* 359. Zoomed in regions of centroid spectrum affirming that background ions can be incorrectly identified as "diagnostic" ions for RvD5 (red arrows).

Supplementary Table 1. From a total of 55 chromatograms, only 16 appear to be usable with S/N >5, while another 5 have S/N >3 so are potentially detected but not quantifiable. Of the 16, 3 don't have good enough standard traces to confirm retention time.

1. For all standards, injected amounts were so low that the MS/MS spectra are extremely poor quality, comprising a significant level of background noise ions. Due to this, the spectra are not useful when matching to sample spectra
2. SPM are denoted by stereospecific names in the study, however reverse phase LC is unable to distinguish enantiomers. It is considered more accurate to denote lipids using annotation that accurately describes their level of annotation, e.g. the lipid being called PD1 or PDX can be called instead 10,17-dihydroxydocosahexaenoic acid

3. Only one chromatogram is shown for each lipid, thus it is impossible to determine the quality of the remaining data, and whether a lipid present in one sample is also present in other patient samples remains unknown.
4. For several pairs of lipids, elution is only 0.1 min apart but it is claimed that both lipids are present, even though only one peak is seen in samples: PD1 and PDX, RvD3 and 17R-RvD3, RvD1 and 17R-RvD1, Mar1 and 7S,14S-diHDHA.

**Lipid**	**noise height**	**peak height**	**Signal to Noise**	**Usable peak, based on S/N>5 (yes) or >3 (borderline)**	**Additional notes on peak quality**
RvD1	0.5	3.35	6.700	YES	For RvD1 and 17R-RvD1, different MRM transitions were used, but both these MRM will detect both lipids, as they are common fragments. However, in samples, only one peak is seen despite both lipids being reported and eluting only 0.2 min apart. There should be two overlapping or closely eluting peaks in patient samples. Standard peak is broader.
RvD2	1.65	2.375	1.439	NO	
RvD3	1	2.5	2.500	NO	Peak shapes are OK. RvD3 and 17R-RvD3 have almost the same retention time so how do they know which is being measured in patient samples? A single peak is seen in patient samples. There should be two overlapping or closely eluting peaks in patient samples if both lipids are present.
RvD4	0.5	2.95	5.900	YES	Sample peak is much broader then standard peak
RvD5	0.9	2.65	2.944	NO	Peak shape is OK
RvD6	1.2	1.9	1.583	NO	Comprises only baseline noise, and appears to have only 1 data point max.
17R-RvD1	1.1	1.35	1.227	NO	Sample peak is very narrow when compared with standard peak, and appears to be only noise with only one data point above baseline, if any. For RvD1 and 17R-RvD1, different MRM transitions were used, but both will detect both lipids, as they are common fragments. However, in samples, only one peak is seen despite both lipids being reported and eluting only 0.2 min apart
17R-RvD3	1.3	2.55	1.962	NO	RvD3 and 17R-RvD3 have almost the same retention time so how do they know which is being measured in patient samples? The patient sample shows a peak that looks like it has only one data point, and is not sufficiently above baseline.

PD1	1	2.7	2.700	NO	Standard peak is much broader. PD1 and PDX have almost the same retention time so how do they know which is being measured in patient samples? A single peak is seen in patient samples so how do they know which this is?
17R-PD1		0	0.000	NO	It was not possible to estimate the S/N based on poor data quality. Peak shape looks OK, but trace is extremely noisy.
PDX	1.5	2.65	1.767	NO	Peak of sample is poor. PD1 and PDX have almost the same retention time so how do they know which is being measured in patient samples? A single extremely poor spike/peak is suggested to be present.
22-OH-PD1	0.3	3.65	12.167	YES	Standard peak looks very narrow and not enough was injected to get a decent confirmation of retention time
PCTR1	2.4	2.7	1.125	NO	Sample peak looks not homogenous and hardly rises above baseline. Very small standard peak.
PCTR2	1.6	3.3	2.063	NO	Very narrow sample peak compared to standard, and hardly rises above baseline
PCTR3	0.8	3.9	4.875	borderine	Above LOD but below LOQ
Maresin1		0	0.000	NO	It was not possible to estimate the S/N based on poor data quality and no actual peak is visible above noise. Mar1 and 7S,14S-diHDHA have almost the same retention time so how do they know which is being measured in patient samples? A single peak is suggested to be present in patient samples so how do they know which this is?
Maresin2	0.05	0.525	10.500	YES	Insufficient standard injected to determine RT, when baseline is going up and down a lot. Sample peak looks reasonable.
22-OH-Maresin1	0.8	1.8	2.250	NO	Insufficient standard injected to determine RT. Peak shapes are very different. Sample peak too low above baseline
14-oxo-Maresin1		0		NO	How did they determine the first peak was the standard, it's smaller than the one after, it was also not possible to calculate S/N due to poor peak quality in the sample
7S,14S-diHDHA		0	0.000	NO	It was not possible to estimate the S/N based on poor data quality. Sample peak is clearly not homogenous (pronounced front shoulder). Mar1 and 7S,14S-diHDHA have almost the same retention time so how do they know which is being measured in patient samples? A single peak is seen in patient samples so how do they know which this is?
4S,14S-diHDHA	0.5	2.75	5.500	YES	Acceptable peak
MCTR1	0.7	3.85	5.500	YES	Strange straight looking sample peak which is very narrow when compared with the standard.

MCTR2	1.8	1.5	0.833	NO	
MCTR3	0.5	3.95	7.900	YES	Pleak looks broader than standard peak, as if there is a shoulder (another lipid) co-eluting
RvT1	0.6	1.2	2.000	NO	Difficult to determine S/N due to very unstable baseline
RvT2	2.2	2.8	1.273	NO	Sample peak is very narrow and insuffiently above noise
RvT3	0.9	3.15	3.500	borderine	Sample peak is not homogenous, and is broad, but achieves LOD but not LOQ
RvT4	2	2.4	1.200	NO	Sample peak looks very narrow and isn't visible above background noise
RvD1, n3DPA	0.4	4	10.000	YES	Acceptable peak
RvD2, n3DPA	1.8	2.7	1.500	NO	Sample peak is not sufficiently above noise
RvD5, n3DPA	1.5	2.85	1.900	NO	Sample peak is not sufficiently above noise
PD1, n3DPA	1.8	1.8	1.000	NO	PD1,n3DPA and 10S,17S-diHDPA have almost the same retention time so how do they know which is being measured in patient samples? A single peak is seen in patient samples so how do they know which this is? The sample peak is in the noise.
10S,17S-diHDPA	1	2.7	2.700	NO	PD1,n3DPA and 10S,17S-diHDPA have almost the same retention time so how do they know which is being measured in patient samples? A single peak is seen in patient samples so how do they know which this is? A peak is seen but doesn't reach high enough to achieve LOD or LOQ
Maresin1, n3DPA	1.4	2.6	1.857	NO	Peak doesn't reach above baseline to be clearly visible
RvE1	0.9	3.05	3.389	borderine	Above LOD but below LOQ
RvE2	0.7	3.25	4.643	borderine	Above LOD but below LOQ
RvE3	0.2	1.4	7.000	YES	Standard peak has a broad base. Sample peak is co-eluting with another lipid but achieves LOQ as baseline appears low
LXA4	1.6	1.9	1.188	NO	LXA4 and 15R-LXA4 have almost the same retention time so how do they know which is being measured in patient samples? A single peak is seen in patient samples so how do they know which this is? The sample peak is within the noise and hardly visible.
LXB4	1.1	3.05	2.773	NO	Insufficient standard injected to determine RT. Sample peak is visible but not above LOD
5S,15S-diHETE	2.4	1.4	0.583	NO	Insufficient standard injected to determine RT. Very narrow sample peak has been integrated which is within the noise

15R-LXA4	0.9	2.35	2.611	NO	Insufficient standard injected to determine RT properly. LXA4 and 15R-LXA4 have almost the same retention time so how do they know which is being measured in patient samples? A single peak is seen in patient samples so how do they know which this is? Sample peak below LOD
15R-LXB4	1.2	3	2.500	NO	How was it determined that the first peak was the standard, it's smaller than the one after? Sample peak below LOD
LTB4	0.3	3.55	11.833	YES	Acceptable peak
5S,12S-diHETE	1.3	1.35	1.038	NO	Poor peak in sample, not above background noise, too little standard injected to determine RT
6-trans-LTB4	0.05	0.525	10.500	YES	Sample peak appears acceptable, as relatively stable baseline can be seen.
12-epi-6-transLTB4		0	0.000	NO	Can't identify RT of standard, but sample has a peak
20-OH-LTB4	0.7	3.45	4.929	borderine	Above LOD but below LOQ
20-COOH-LTB4	0.4	3.8	9.500	YES	Can't identify RT of standard, but sample has a peak
LTC4	0.2	2.6	13.000	YES	Acceptable peak
LTD4	0.3	3.75	12.500	YES	Acceptable peak
LTE4	0.4	3.5	8.750	YES	Acceptable peak
PGD2	0.3	2.65	8.833	YES	Acceptable peak
PGE2	0.3	2.85	9.500	YES	Acceptable peak
PGF2a		0	0.000	NO	Impossible to determine baseline, peak below LOD
TXB2		0	0.000	NO	Peak is weak and does not look like the characteristic keto-enol tautomers

References

1 https://www.ema.europa.eu/en/ich-q2-r1-validation-analytical-procedures-text-methodology.

2 https://www.who.int/medicines/areas/quality_safety/quality_assurance/28092018Guideline_Validation_AnalyticalMethodValidation-Appendix4_QAS16-671.pdf.

3 http://www.pharmacopeia.cn/v29240/usp29nf24s0_c1225.html.

4 Heyden, Y. V. B., R. *The limit of detection*. Vol. 22 82-55 (LCGC Europe, 2009).

5 Wenzl, T., Haedrich, J, Schaechtele, A, Robouch, P, Stroka, J. Guidance Document on the Estimation of LOD and LOQ for Measurements in the Field of Contaminants in Feed and Food. *EUR 28099; Publications Office of the European Union: Luxembourg,* **https://publications.jrc.ec.europa.eu/repository/bitstream/JRC102946/eur%2028099%20en_lod%20loq%20guidance%20document.pdf** (2016).
6 Araujo, P. Key aspects of analytical method validation and linearity evaluation. *J Chromatogr B Analyt Technol Biomed Life Sci* **877**, 2224-2234, doi:10.1016/j.jchromb.2008.09.030 (2009).
7 Desimoni, E., Brunetti, B. Signal to noise ratio; Limit of detection; Standard error of the regression; Chromatographic/Voltammetric/Spectroscopic signals. *PHARMACEUTICA ANALYTICA ACTA* **6**, doi:http://dx.doi.org/10.4172/2153-2435.1000355 (2015).
8 Commission, E. 2002/657/EC: Commission Decision of 12 August 2002 implementing Council Directive 96/23/EC concerning the performance of analytical methods and the interpretation of results (Text with EEA relevance) (notified under document number C(2002) 3044) *https://op.europa.eu/en/publication-detail/-/publication/ed928116-a955-4a84-b10a-cf7a82bad858/language-en* (2002).
9 Research, C. f. D. E. a. & Research, C. f. B. E. a. https://www.fda.gov/regulatory-information/search-fda-guidance-documents/analytical-procedures-and-methods-validation-drugs-and-biologics. *https://www.fda.gov/regulatory-information/search-fda-guidance-documents/analytical-procedures-and-methods-validation-drugs-and-biologics* (2015).
10 Olivieri A, F. N., Ferre J, Boque R, Kalivas JH, Mark H. UNCERTAINTY ESTIMATION AND FIGURES OF MERIT FOR MULTIVARIATE CALIBRATION, IUPAC Technical Report. *Pure Appl Chem* **78**, 633-661, doi:10.1351/pac200678030633.
11 FDA, U. *Bioanalytical Method Validation Guidance for Industry*.
12 Colas, R. A., Gomez, E.A., Dalli, J. *Methodologies and Procedures Employed in the Identification and Quantitation of Lipid Mediators via LC-MS/MS* (Protocol Exchange, 2020).
13 Dalli, J., Colas, R. A., Walker, M. E. & Serhan, C. N. Lipid Mediator Metabolomics Via LC-MS/MS Profiling and Analysis. *Methods Mol Biol* **1730**, 59-72, doi:10.1007/978-1-4939-7592-1_4 (2018).
14 Sinha, G. Critics challenge data showing key lipids can curb inflammation. *Science* **376**, 565-566, doi:10.1126/science.abq8439 (2022).

Supplementary Figure 1

A

Chromatogram showing elution of LTB3 standard occurs between 11.35 and 11.5 min

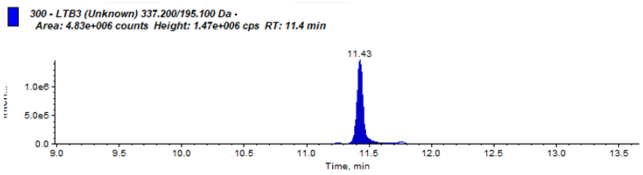

Three blank methanol injections showing no peak. Integration of the window (11.25-11.5) all show areas above 2000

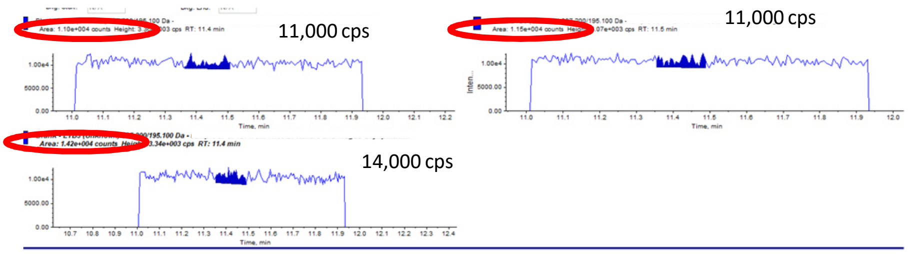

B

Chromatogram showing elution of 8-HETE standard occurs between 13.95-14.08

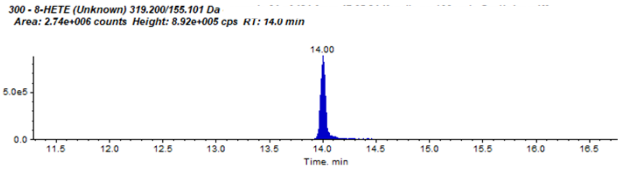

Three blank methanol injections showing no peak. Integration of the window (13.95-14.08) all show areas above 2000

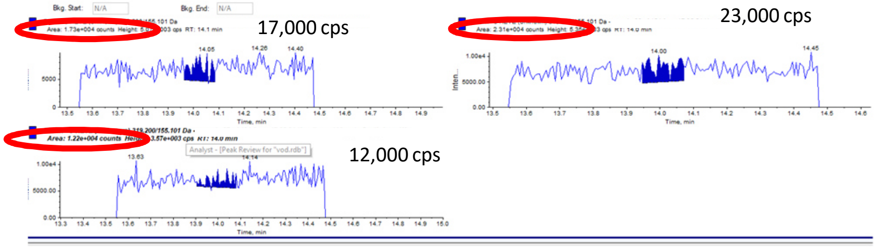

C

Chromatogram showing elution of 15-HETE standard occurs between 13.5-13.65

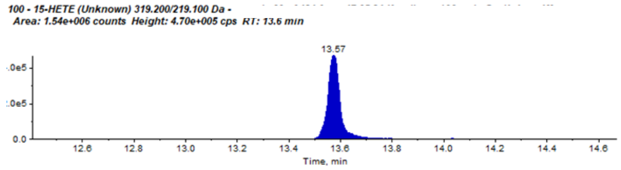

Two blank methanol injections showing no peak. Integration of the window (13.5-13.65) all show areas above 2000

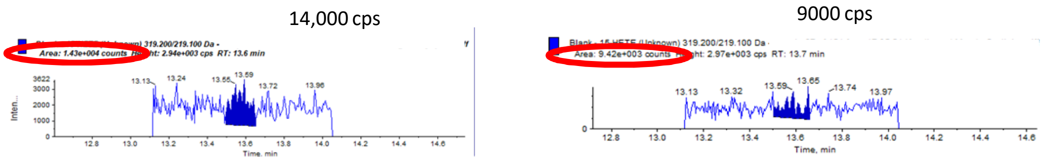

Supplementary Figure 2

A

Chromatogram showing elution of 5-HETE standard occurs between 14.3 and 14.4 min

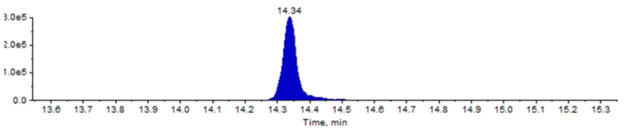

A blank methanol injection showing no peak/noise. Integration of the window (14.3-14.4) shows area above 2000

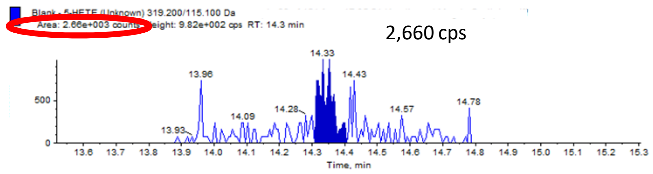

B

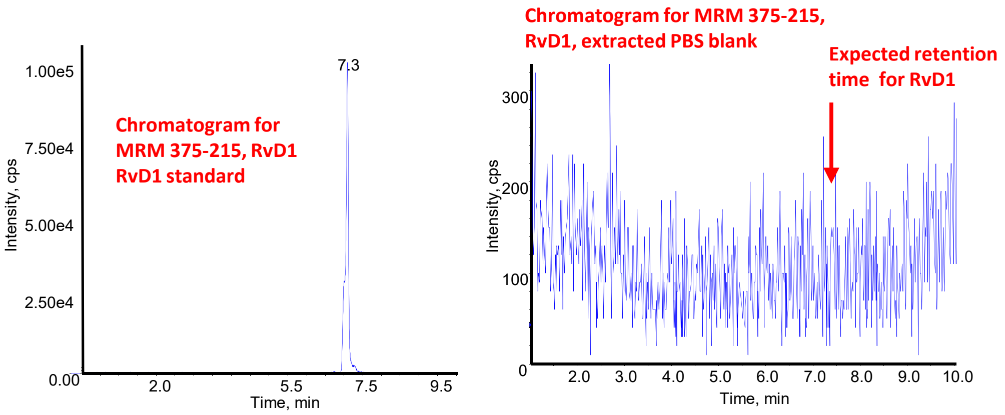

C

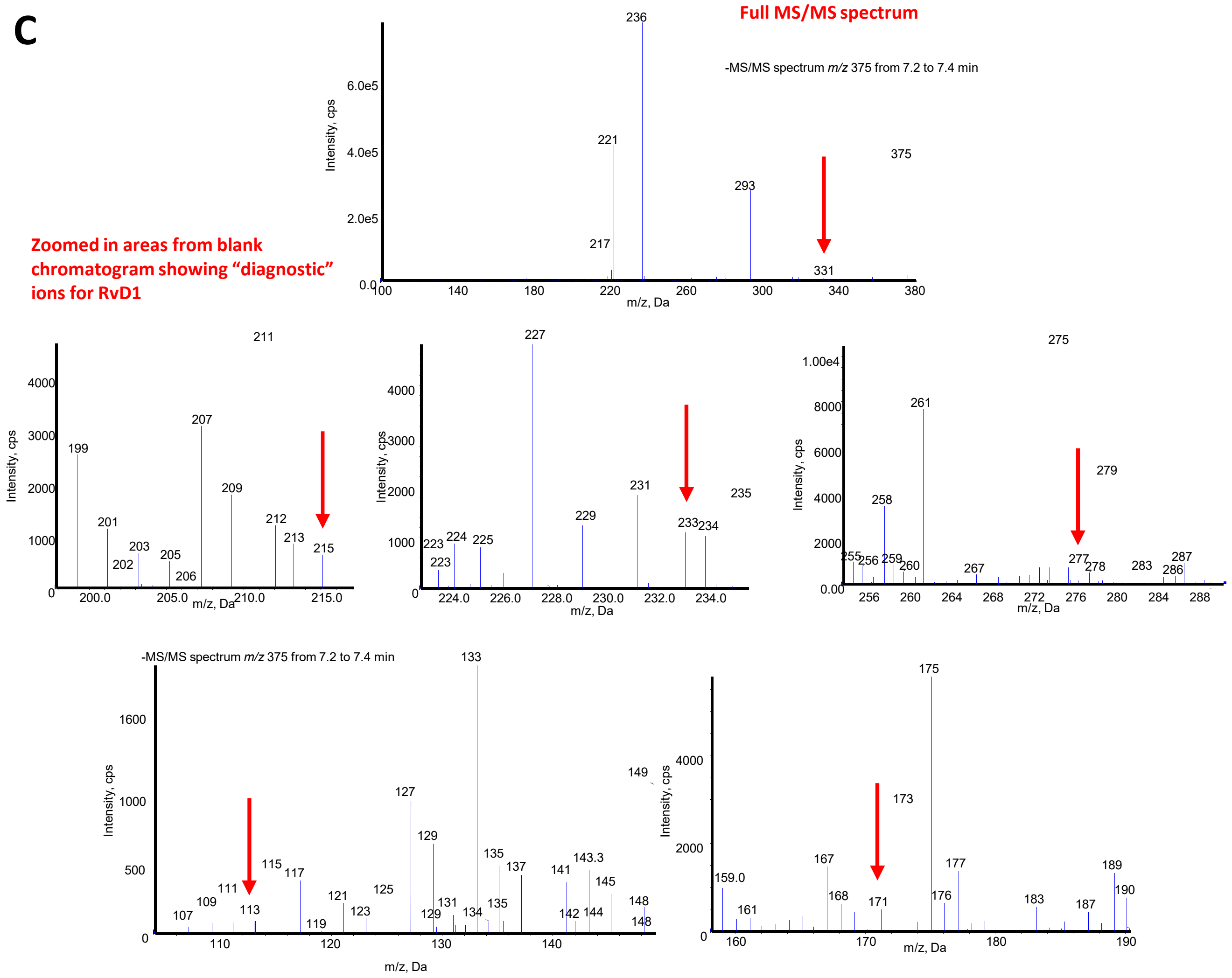

Supplementary Figure 3

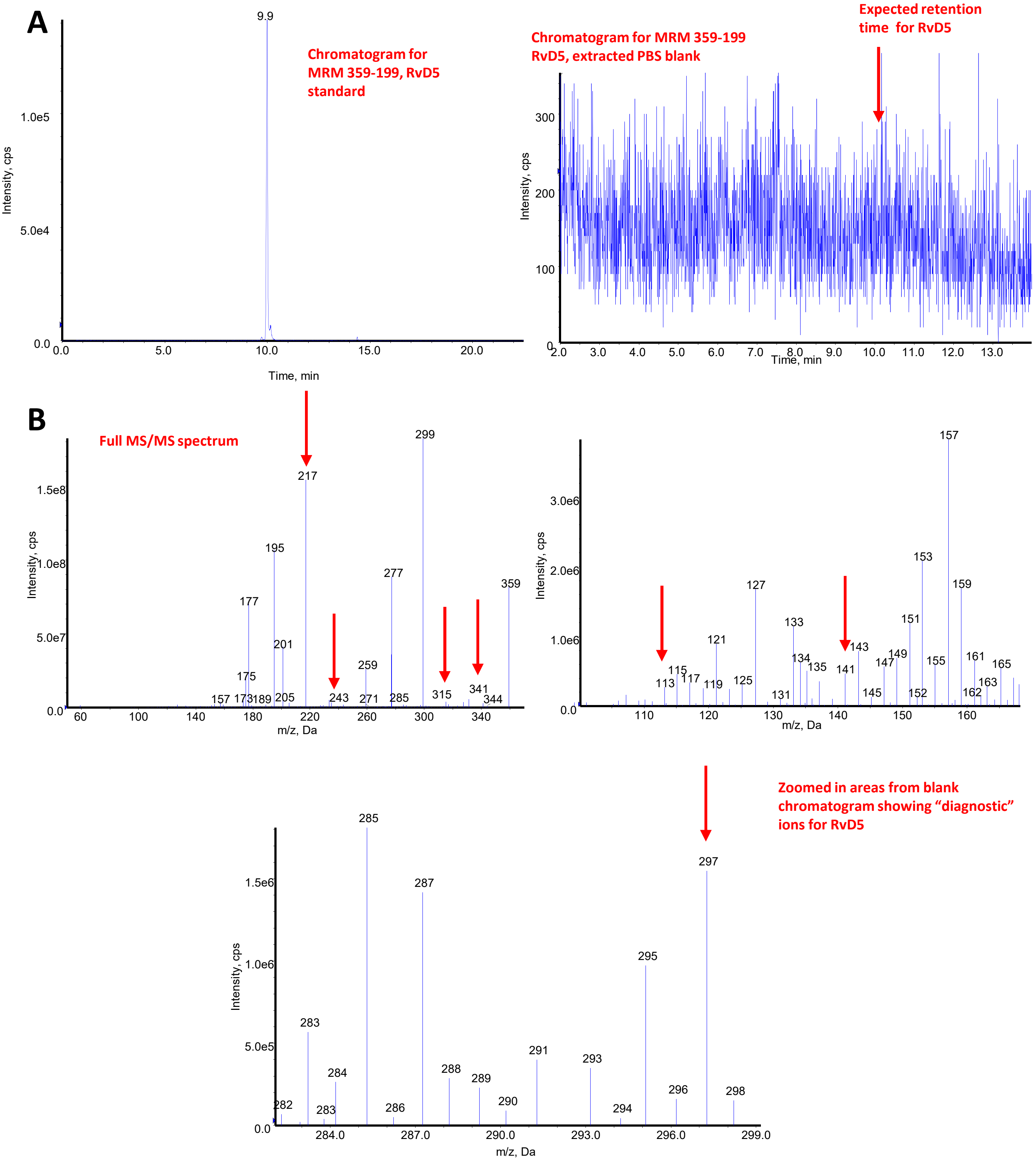

Supplement: Supplementary file 1 — Supplementary Information [file 41467_2023_41766_MOESM1_ESM.pdf]
